# Combination therapy using GDNF and cell transplant in Parkinson’s disease

**DOI:** 10.1186/s13024-022-00553-9

**Published:** 2022-07-16

**Authors:** Bin Xiao, Su-Chun Zhang, Eng-King Tan

**Affiliations:** 1 Department of Neurology, National Neuroscience Institute, Singapore, Singapore; 2 Waisman Center, University of Wisconsin-Madison, Madison, WI USA; 3 Department of Neuroscience, Department of Neurology, School of Medicine and Public Health, University of Wisconsin, Madison, WI USA; 4 Neuroscience and Behavioral Disorders Program, Duke-NUS Medical School, Singapore, Singapore

**Keywords:** GDNF, Cell therapy, Parkinson’s disease, Innervation, Neural circuit

Cell replacement therapy is a viable option for Parkinson’s disease (PD) due to a selective loss of dopaminergic (DA) neurons in the substantia nigra (SN). In the past, cell therapy in PD utilized midbrain tissue of aborted fetuses, but despite symptomatic relief in some patients, ethical concerns and troubling side effects dampen widespread clinical application [1]. The advancement of stem cell technology, especially recent progress in generating authentic midbrain DA (mDA) neurons from human pluripotent stem cells (hPSCs), has led to a resurgence of interest in autologous cell transplantation [2].

Some of the major issues influencing the efficacy of PD cell transplantation lie in the degree of target innervation and regulation of dopamine release. Most grafts have been seeded in the striatum region to ensure sufficient proximal innervation. Motor functions can be improved, as DA neurons are autonomous pacemakers which tonically release dopamine often at the non-synaptic terminals. Several protocols have been established to direct the differentiation of hPSCs to mDA neurons, including a recent one for the generation of authentic A9 DA neurons with pacemaker activity which showed robust survival and axon growth in a rat PD model and rescued its motor deficits [3]. However, mDA neurons innervate the striatum and release dopamine to fine-tune the voluntary movement orchestrated by intricate neural circuits. Endogenous substantia nigra DA neurons receive regulatory input from diverse sources. Without correct afferents, the ectopically grafted DA neurons would not be able to properly reconstruct the neural circuits. Such defective integration due to ectopic transplantation may contribute to the adverse effects, like dyskinesia, seen in the fetal midbrain transplanted patients, which is a major hurdle for the application of cell therapy in PD. SN transplantation shows promise to solve the above problem, with graft-derived axons extending and innervating the caudate-putamen as well as receiving appropriate synaptic inputs in mice [4]. However, such a strategy encounters a major hurdle when translating to patients in which the SN-striatum distance is nearly 10 times that in mice. There is therefore a critical need to develop means to promote axonal growth from grafted mDA neurons.

One of the ways to enhance the growth of axons from the grafted mDA neurons is the use of glial cell line-derived neurotrophic factor (GDNF). Applications of GDNF to promote the survival and regeneration of endogenous DA neurons to compensate for the loss of neurons in PD are clinically relevant. Despite increases in ^18^F-DOPA uptake on positron emission tomography (PET) imaging reported in many clinical trials with GDNF, no significant improvement in motor function has been observed in the participants in placebo-controlled trials, possibly in part due to the inability of GDNF to improve the health of morbid DA neurons in PD patients [5].

Recognizing the inability of grafted cells in SN to sufficiently innervate and the potential of GDNF to support the growth of the nascent DA neurons, Moriarty et al. hypothesized that GDNF can promote the function of the grafted neurons in SN [6]. In their study, GDNF was delivered via adeno-associated viruses (AAVs) in the striatum prior to or following the transplantation of DA neurons into SN. The authors utilized a PITX3-GFP reporter line to trace the growth and innervation of DA neurons. They found that GDNF facilitated better connectivity of grafted DA neurons to striatum and other targets that are normally innervated by endogenous mDA neurons, resulting in improved striatal dopamine levels and restoration of motor functions. In addition, GDNF selectively induced mDA neuron growth but not non-mDA neuronal growth. The innervation regions by mDA axons matched the distribution of GDNF. These observations suggest that GDNF is able to efficiently enable grafted mDA to function properly in the experimental model (Fig. 1A).

**Fig. 1:**
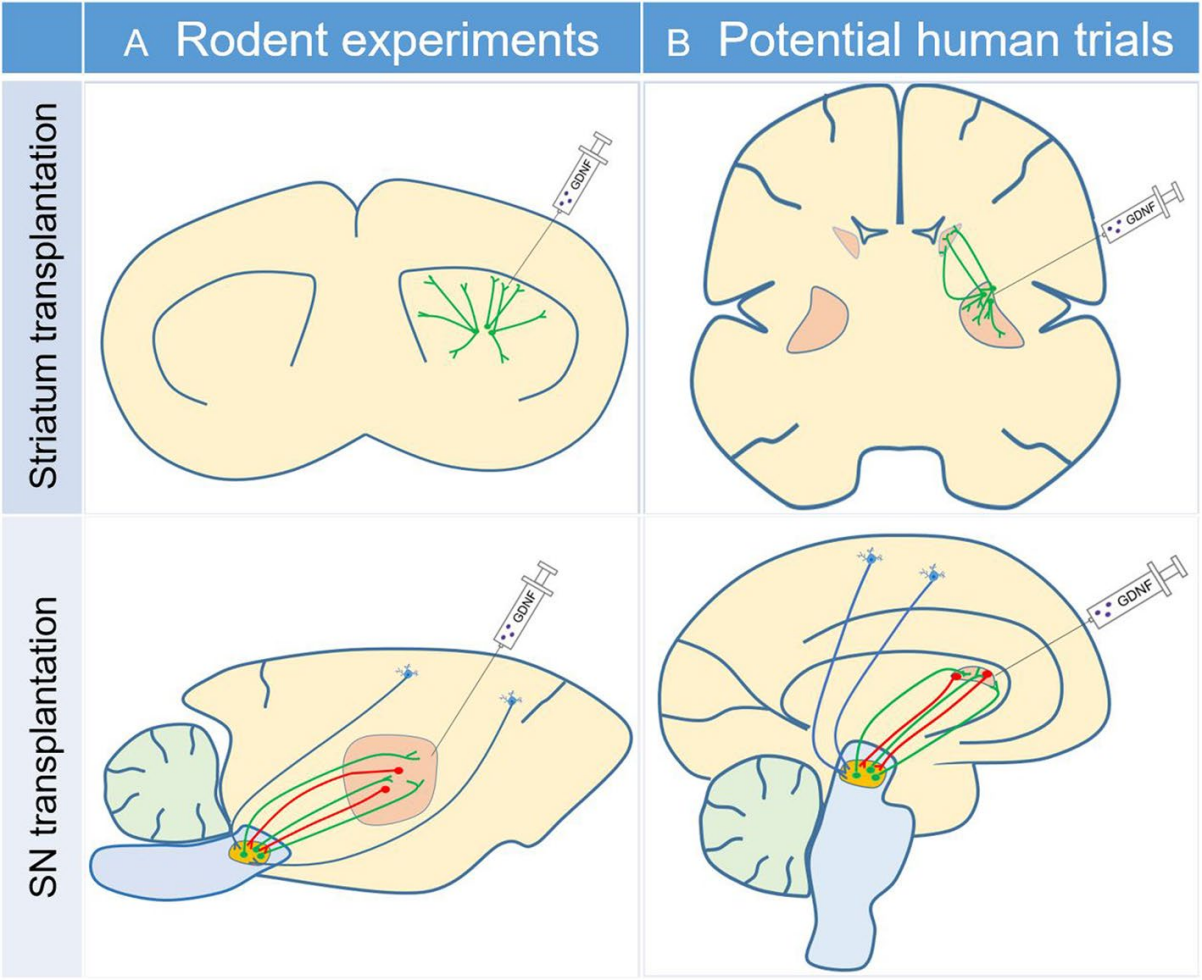
Combination therapy of glial derived neurotrophic factor (GDNF) and cell transplant in substantia nigra (SN) and/or striatum may represent a potential therapeutic option for Parkinson’s disease (PD). (A) Moriarty et al. found that striatum and other targets that are normally innervated by endogenous mDA were densely and specifically innervated by the grafted DA neurons upon addition of GDNF, resulting in clinical improvement in a rat PD model. (B) Future clinical trials can potentially explore a combination of GDNF and cell transplantation in SN and/or striatum to evaluate if GDNF supports the survival and induces axonal branching of the striatal graft and if it induces more branching of the DA axons towards the site of GDNF for SN transplant. Green fibers denote efferent innervation of the striatum by the grafted DA neurons; red fibers denote afferent innervation of the grafted neurons by the host’s striatal neurons; blue fibers denote afferent innervation of the grafted neurons by the host’s cortex and other brain regions.

The study can potentially help to bridge the current limitations and outcomes in cell transplantation and GDNF based therapeutics. Although GDNF failed in most clinical trials, it is generally safe when applied locally. The prospect of using GDNF in combination with cell transplantation is exciting. Further validation of the authenticity of DA neurons and their functions utilizing more sophisticated experimental methods, including electrophysiology and PET, and demonstration of the efficacy of motor symptom restoration utilizing more comprehensive behavior assays will provide more robust support to the authors’ findings. In the recent study of cell therapy in Parkinsonian monkeys, neuronal fibers extended over 2,400 µm away from the graft, but their density decreased with the distances to the graft [7]. It will be interesting to determine if GDNF supports the survival and induces axonal branching of the striatal graft. In addition, in a recent case of autologous transplantation in the striatum, gradual clinical improvement was observed during the 18 to 24 months after implantation, consistent with the time frame for DA neuron innervation [2]. It is conceivable that it would take much longer for the transplantation in the SN to innervate the striatum area with a distance of a few centimeters from the graft site. Studies are warranted to examine if GDNF induces nigral transplant to sufficiently extend axons towards the striatum where GDNF is delivered and the duration it will take for the graft innervation and neural circuit reconstruction in nonhuman primate models or in humans (Fig. 1B). If the improvement is significant and provides better benefit than levodopa treatment, the prodromic or premotor stage in PD may provide an ideal treatment window for cell therapy to allow the graft to innervate and repair the damaged neural circuits. Cell therapy trials combined with GDNF should also consider selecting the more “benign” patients with slow progression and lesser non-motor symptoms to ensure that the potential benefits overweigh the risks.

## Data Availability

Not applicable.

## Abbreviations

PD: Parkinson’s disease
DA: Dopaminergic
hPSCs: Human pluripotent stem cells
SN: Substantia nigra
mDA: Midbrain DA
GDNF: Glial cell line-derived neurotrophic factor
PET: Positron emission tomography
AAVs: Adeno-associated viruses
